# Melanoma of unknown primary origin with skeletal muscle metastasis: a case report

**DOI:** 10.1186/s13256-023-03813-4

**Published:** 2023-03-12

**Authors:** Ny Ony Tiana Florence Andrianandrasana, Rova Malala Fandresena Randrianarisoa, Patty Navoly, Mirana Andoniaina Christiana Ranaivoson, Hanta Marie Danielle Vololontiana, Florine Rafaramino

**Affiliations:** 1 Oncology Department, Joseph Ravoahangy Andrianavalona Hospital, Antananarivo, Madagascar; 2 Department of Internal Medicine, Joseph Raseta Befelatanana Hospital, Antananarivo, Madagascar

**Keywords:** Biopsy, Melanoma, Melanoma of unknown primary origin, Muscle metastasis

## Abstract

**Background:**

Melanoma is usually discovered from an irregular skin patch or a modification of a preexisting patch. Cutaneous and lymph node metastases are common. Muscle metastases are rare. We report a case of melanoma with infiltration of the gluteus maximus, which had normal dermatological examination.

**Case presentation:**

A 43-year-old Malagasy man with no history of skin surgery was admitted with progressively worsening dyspnea. On admission, he presented with superior vena cava syndrome, painless cervical lymphadenopathy, and a painful swelling in the right buttock. Skin and mucous membrane examination did not reveal any abnormal or suspicious lesions. The biology was limited to a C-reactive protein of 40 mg/L, a white blood cell count of 23 G/L, and a lactate dehydrogenase level of 1705 U/L. The computed tomography scan showed several lymphadenopathies, compression of the superior vena cava, and a tissue mass at the expense of the gluteus maximus. Cervical lymph node biopsy and cytopuncture of the gluteus maximus were consistent with a secondary melanoma location. A stage IV melanoma of unknown primary origin, and with stage TxN3M1c associated with lymph node metastases and extension to the right gluteus maximus, was suggested.

**Conclusions:**

Melanoma of unknown primary origin accounts for 3% of diagnosed melanomas. Diagnosis is difficult in the absence of a skin lesion. Patients are diagnosed with multiple metastases. Muscle involvement is unusual and may suggest a benign pathology. In this context, biopsy remains essential for diagnosis.

## Background

Melanoma is one of the most aggressive forms of skin cancer, accounting for about three-quarters of all deaths. Over the past two decades, the incidence of melanoma has been steadily increasing [1]. According to the Global Cancer Observatory, the incidence was 3.4 cases per 100,000 population in 2020, with 57,043 deaths [2].

The diagnosis of melanoma is usually made in the presence of an irregular skin patch, or a change in a preexisting patch. The diagnosis is then based on histological examination. Cutaneous and lymph node metastases are common. Hematogenous spread is possible, often involving the lungs, liver, and brain [3]. Striated muscle metastases are unusual [4].

In the absence of a skin lesion, melanoma represents a diagnostic challenge that can delay therapeutic management. We report a case of melanoma with infiltration of the right gluteus maximus, which had a normal dermatological examination. Our objectives are to discuss the frequency, pathophysiological mechanism, and prognosis of this type of melanoma in relation to the literature.

## Case presentation

A 43-year-old man of Malagasy nationality with phototype V (Fitzpatrick) skin was admitted to the oncology department for dyspnea, which had been evolving for a few weeks with progressive worsening. He reported dysphonia, dysphagia, and associated episodes of dry cough. He had asthenia and weight loss without fever. He was followed by the thoracic surgery team for a right cervical lymphadenopathy, which had been evolving for 4 months and for which the histological result of the biopsy was pending.

He had a history of alcoholism and chronic weaned smoking. He had no history of skin pigmentation or skin surgery, and no personal or family history of cancer.

**Fig. 1:**
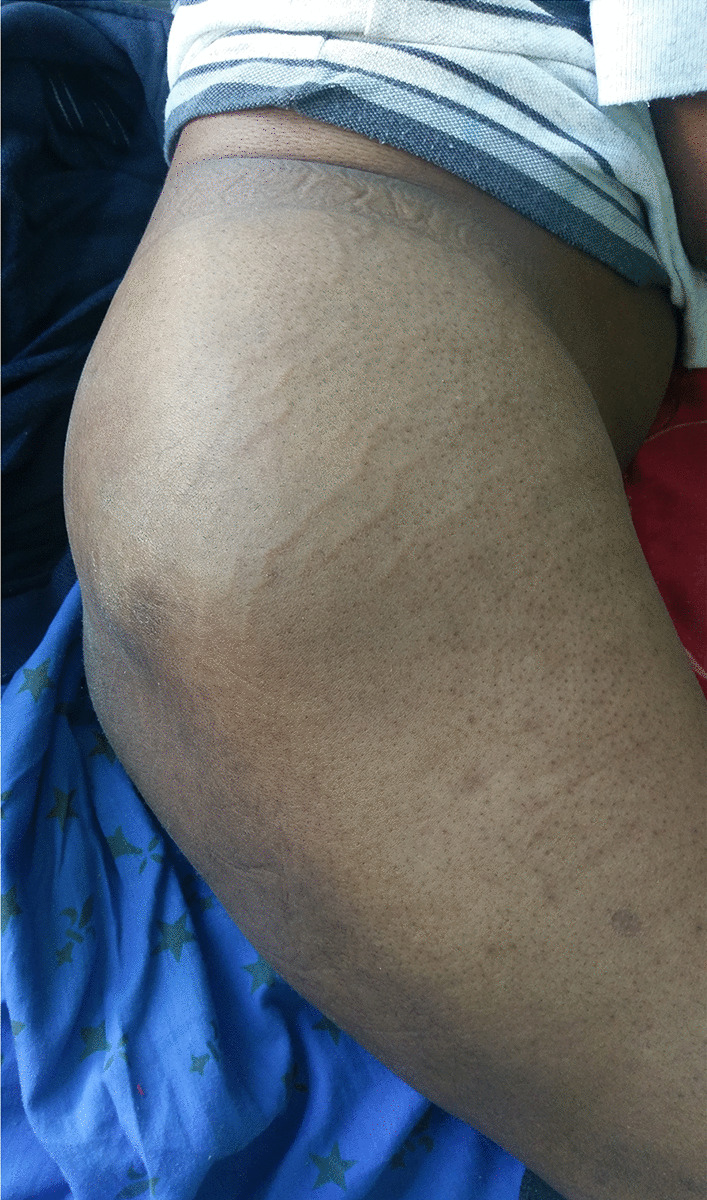
Muscle swelling in the patient’s right buttock

On admission, hemodynamic parameters were stable. General condition was impaired with a World Health Organization performance status score of 3. Examination revealed facial edema and venous collateral circulation in the neck. He had painless right cervical lymphadenopathy and a swelling in the right buttock that was painful to palpation and firm in consistency (Fig. 1). Careful examination of the skin and mucous membranes did not reveal any abnormal or suspicious lesions. The rest of the examination was normal.

The blood count showed a white blood cell count of 23 g/L with a predominance of neutrophils. The C-reactive protein was 40 mg/L. Serum ionogram, calcium, and creatinine levels and liver function tests were normal. Human immunodeficiency virus (HIV) and hepatitis B and C serologies were negative. The lactate dehydrogenase level was 1705 U/L. The carcinoembryonic antigen test was negative.

**Fig. 2:**
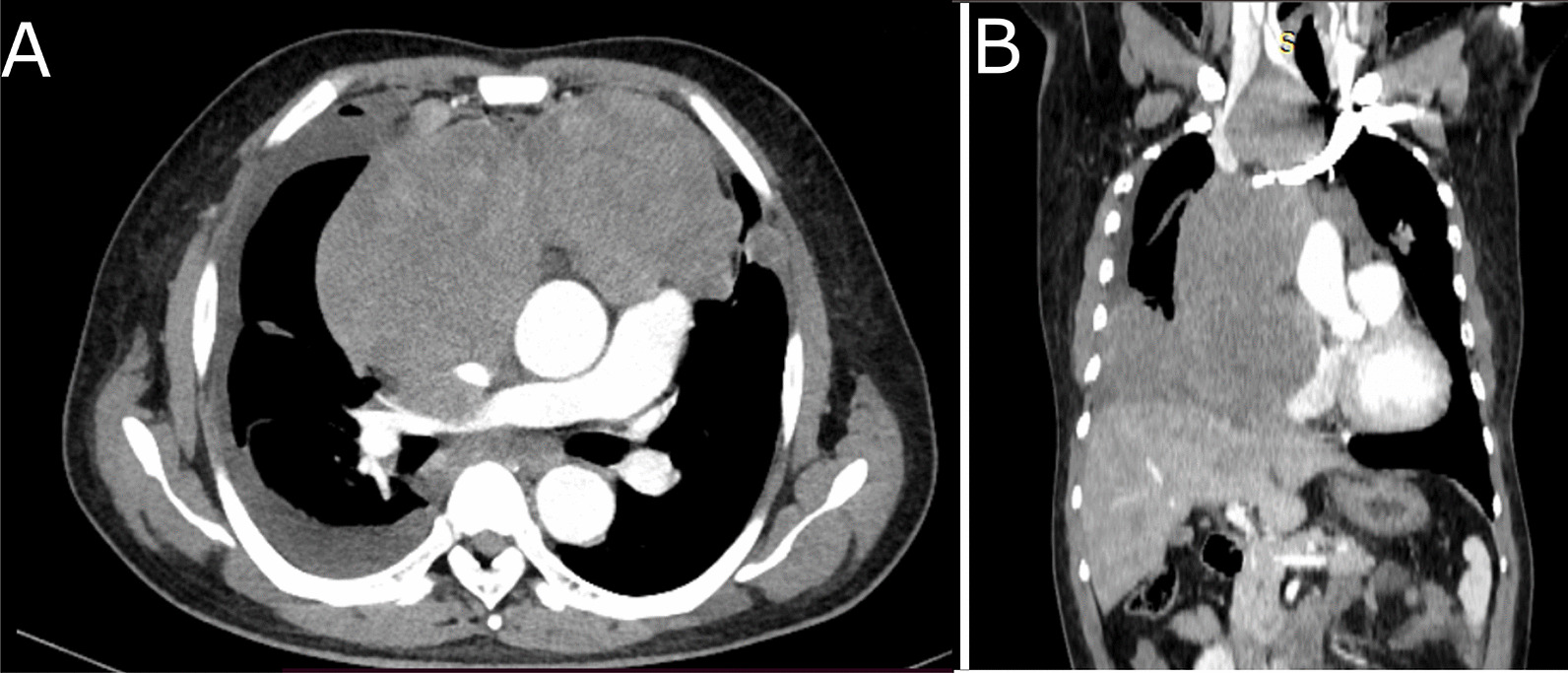
CT scan with injection in axial (**A**) and coronal (**B**) sections. Mediastinal lymph node complex and compression of the right superior vena cava

**Fig. 3:**
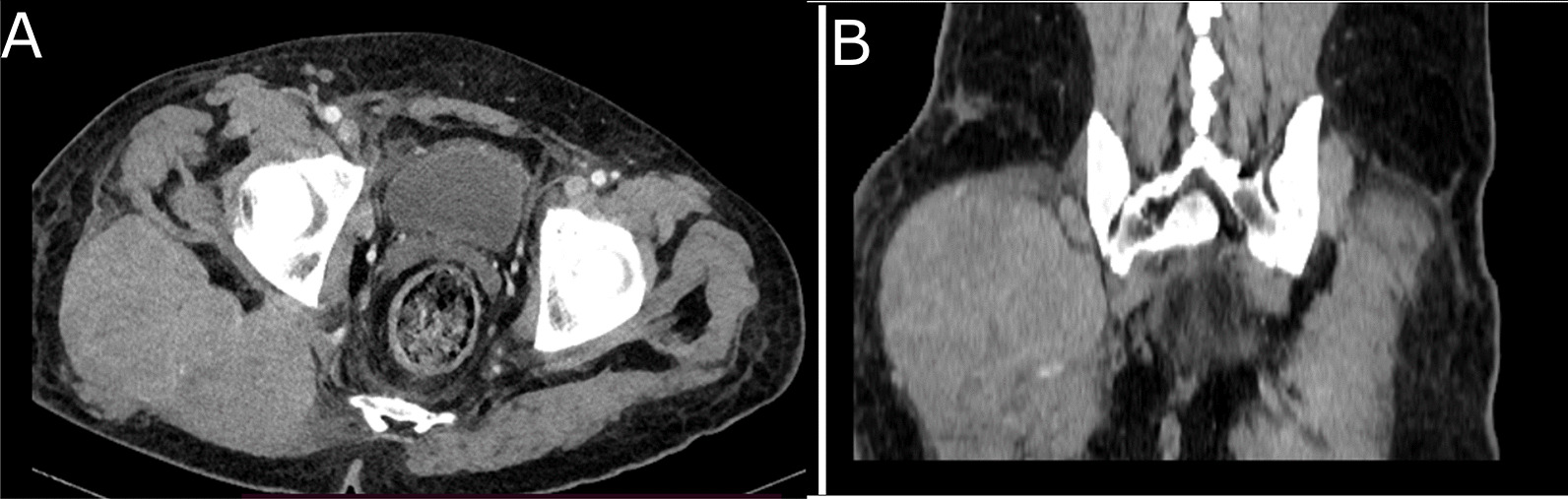
CT scan in axial (**A**) and coronal (**B**) sections. Mass at the expense of the gluteus maximus muscle

The thoracic-abdominal-pelvic computed tomography scan revealed cervical, mediastinal, and retroperitoneal lymphadenopathy and compression of the superior vena cava (Fig. 2). The mass in the right buttock was well-limited and heterogeneous, measuring 63 × 127 mm, and was located at the expense of the gluteus maximus muscle with low enhancement (Fig. 3).

**Fig. 4:**
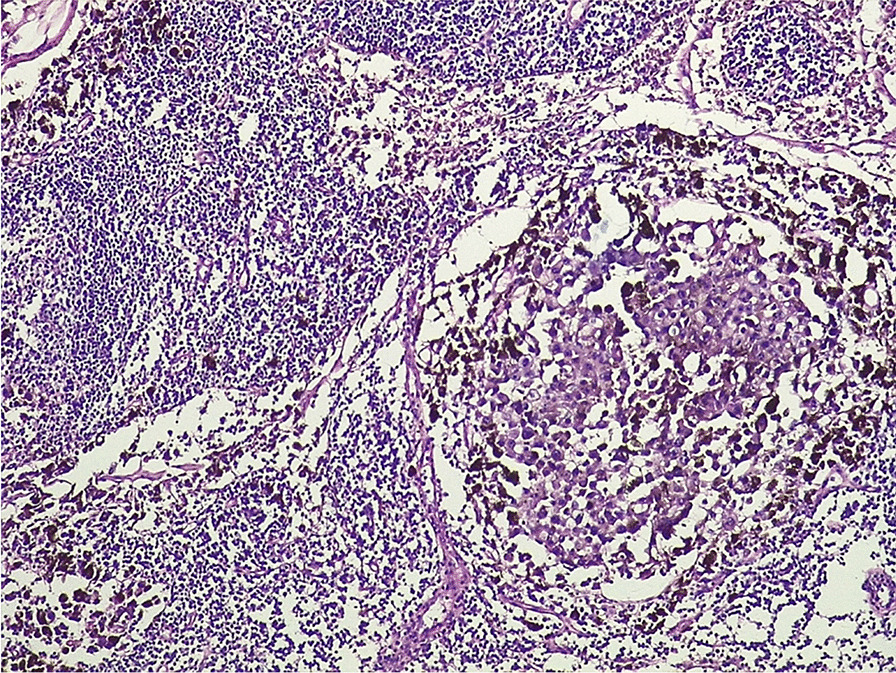
Histology of lymph node biopsy. Cell infiltration and melanin pigments [hematoxylin and eosin (HE) × 40]

The result of the histological examination of the cervical lymph node biopsy showed invasion of the lymph node and capsular architecture by globular cells with abundant cytoplasm, cytonuclear atypia, and melanin pigments (Fig. 4). A cytopuncture of the gluteus maximus was performed and cytology showed an infiltration of neutrophils and macrophages, tattooed with melanin pigments.

*BRAF V600* mutation testing could not be performed due to the technical platform.

A stage IV malignant melanoma with lymph node metastases and extension to the right gluteus maximus was suggested. No primary skin site was identified. This is equivalent to stage TxN3M1c. The dyspnea was related to the superior vena cava syndrome, secondary to lymph node compression. A brain scan was performed and showed no secondary lesions.

Corticosteroid therapy with methylprednisolone and preventive anticoagulation with enoxaparin were taken for superior vena cava syndrome. After multidisciplinary discussion, mediastinal radiotherapy for decompression was started in the first instance. Treatment with dacarbazine was planned, depending on the availability of chemotherapy molecules.

The short-term course was marked by rapid regression of the superior vena cava syndrome and improvement in respiratory symptomatology. No suspicious skin patches appeared during follow-up.

## Discussion and conclusions

In 2019, Andrianarison et al. reported 47 cases of skin cancer over an 8-year period in Madagascar. Squamous cell carcinoma was predominant, followed by melanoma, found in 17 cases (37%) and 10 cases (21%), respectively [5]. The rarity of melanoma is explained by the limited number of diagnostic and therapeutic centers, underestimating the data. It is also explained by the darker skin (phototype V and VI) of the Malagasy population, making them less likely to develop melanoma.

Melanoma is an aggressive melanocyte tumor with easy and early metastasis. In the case of a metastatic melanoma, a primary lesion must be rapidly identified to allow tumor classification and therapeutic orientation. Assessment should include a full dermatological examination and anorectal, genital, and ophthalmological examination. The primary lesions commonly present as skin, mucous membrane, or ocular lesions. In some cases, it is not identified and is referred to as a melanoma of unknown primary (MUP) origin.

The first entity of MUP was proposed by Das Gupta et al. in 1963 [6]. It is defined by the presence of histologically confirmed melanoma in skin/subcutaneous tissue, lymph nodes, or viscera, without manifestation of a primary lesion. MUP is rare, accounting for approximately 3% of diagnosed melanomas [7]. It usually occurs in people in their 40s and 50s, with a male predominance.

The pathophysiological mechanism of MUP is not fully understood. Two hypotheses have been suggested: complete spontaneous regression of the primary lesion after metastasis and primary origin from ectopic melanocytes in lymph nodes or viscera [6, 8]. The spontaneous regression hypothesis is the most supported, secondary to an immunological response [9].

Metastases from MUP may be subcutaneous, lymph node, or visceral [3]. Lymph node metastases are the most common, occurring in 60% of cases. Muscle metastases are rare and solitary forms occur in 0.8% of cases [4]. This low incidence may be attributed to the hostile environment of the muscles for cancer progression. In small series and single case reports, muscle metastases from melanoma often manifest as a palpable and painful mass. The paravertebral and proximal limb muscles are most affected [4]. Some cases were initially misdiagnosed as a lipoma [10]. However, the certainty of diagnosis is anatomopathological, obtained from a muscle or lymph node biopsy.

Due to the lack of precision in the American Joint Committee on Cancer staging system, the authors classified MUP as stage III or IV [3, 11]. Patients with lymph node, skin/subcutaneous, or soft tissue metastases are classified as stage III. Other forms are classified as stage IV. In our case, the melanoma was classified as stage IV. According to the TNM staging, our case can be classified as TxN3M1c.

The prognosis of MUP has been shown to be better than melanoma of known origin at the corresponding stage [12, 13]. In other studies, the clinical outcome is worse for patients with MUP [8, 14]. These results remain inconclusive as the data are contradictory. Furthermore, treatment depends on the staging of the lesion and is generally similar in both forms of melanoma.

The diagnosis of melanoma is difficult in the absence of a skin lesion. MUP is thus evoked. Patients are diagnosed with subcutaneous, lymph node, or visceral metastases. Invasion of striated muscles is rare in melanoma, but also in cancer in general. Muscle metastases usually present as a painful mass and may suggest a benign pathology. Biopsy plays an important role in the certainty of diagnosis and should be performed systematically for any adenopathy or muscle mass.

## Data Availability

All data generated are included in this published article.

## Abbreviation

MUP: Melanoma of unknown primary
